# Internal hernia after gastrectomy for gastric cancer in minimally invasive surgery era

**DOI:** 10.1007/s10120-019-00931-1

**Published:** 2019-02-13

**Authors:** Kyong Min Kang, Yo Seok Cho, Sa-Hong Min, Yoontaek Lee, Ki Bum Park, Young Suk Park, Sang-Hoon Ahn, Do Joong Park, Hyung-Ho Kim

**Affiliations:** 1 Department of Surgery, Seoul National University Bundang Hospital, 82 Gumi-ro 173beon-gil, Bundang-gu, Seongnam, 13620 South Korea; 2 Department of Surgery, Seoul National University College of Medicine, Seoul, South Korea; 3 Department of Surgery, Korea University Ansan Hospital, Ansan, South Korea; 4 Department of Surgery, Kyungpook National University Hospital, Daegu, South Korea

**Keywords:** Hernia, Gastric cancer, Laparoscopy, Gastrectomy, Risk factors

## Abstract

**Background:**

The incidence and clinical presentation of internal hernia after gastrectomy have been changing in the minimally invasive surgery era. This study aimed to analyze the clinical features and risk factors for internal hernia after gastrectomy for gastric cancer.

**Methods:**

We retrospectively analyzed internal hernia after gastrectomy for gastric cancer in 6474 patients between January 2003 and December 2016 at Seoul National University Bundang Hospital. Multivariable logistic regression was performed to evaluate risk factors.

**Results:**

Internal hernias identified by computed tomography or surgical exploration were 111/6474 (1.7%) and the median interval time was 450 days after gastrectomy. Fourteen (0.9%) of the 1510 patients who underwent open gastrectomy and 97 (2.0%) of the 4964 patients who underwent laparoscopic gastrectomy developed internal hernia. Of the 6474 patients, internal hernia developed in 0 (0%), 9 (1.1%), 40 (3.1%), 56 (3.3%), 6 (2.3%), and 0 (0%) patients who underwent Billroth I, Billroth II, Roux-en-Y, uncut Roux-en-Y, double tract, and esophagogastrostomy reconstructions, respectively. Fifty-nine (53.2%) of 111 patients with symptomatic hernia underwent surgery. Of the 59 internal hernias, treated surgically, 32 (53.2%), 27 (45.8%), and 0 (0%) were identified in jejunojejunostomy mesenteric, Petersen’s, and transverse colon mesenteric defects, respectively. In multivariate analysis, non-closure of mesenteric defects (*P* < 0.01), laparoscopic approach (*P* < 0.01), and totally laparoscopic approach (*P* = 0.03) were independent risk factors for internal hernia.

**Conclusions:**

The potential spaces such as Petersen’s, jejunojejunostomy mesenteric, and transverse colon mesenteric defects should be closed to prevent internal hernia after gastrectomy for gastric cancer.

## Introduction

Laparoscopic gastrectomy has been widely adopted as a treatment for gastric cancer, not only in Eastern Asia but also in Western countries. It is minimally invasive and reduces the risk of postoperative adhesions; thus, enabling earlier recovery of bowel movement and reduced hospital stay. Moreover, several trials have reported that the long-term oncologic outcome of laparoscopic surgery is similar to that of conventional open surgery [1–6].

Gastric cancer surgery includes lymph-node dissection and bowel reconstruction for bowel continuity after gastrectomy. During this process, internal hernia could develop. The incidences of internal hernia were reported from less than 1–5% of patients after open gastrectomy [7–9]. After the adoption of laparoscopic surgery, the incidences have increased to 5–9% [10–14]. Delay in management for internal hernia might result in intestinal strangulation and the subsequent need for bowel resection [4, 14, 15].

Although several studies have reported various complications after gastrectomy for gastric cancer, only a few studies have evaluated late postoperative complications, especially internal hernia [3, 6, 16]. The aim of this study was to analyze the clinical characteristics of internal hernia after introduction of minimally invasive surgeries and to evaluate the risk factors of internal hernia to prevent the life-threatening consequences.

## Materials and methods

From January 2003 to December 2016, 7041 patients underwent gastrectomy for gastric cancer at Seoul National University Bundang Hospital, Seongnam, South Korea. Of these, 567 palliative gastrectomies were excluded because information regarding long-term complications including internal hernia could not be gathered for these patients. Consequently, 6474 patients who underwent curative gastrectomy were retrospectively enrolled in this study.

The study retrospectively analyzed patients with internal hernia identified by surgical exploration or abdominal computed tomography (CT) from surgical and medical records. The median follow-up was 60.3 months after gastrectomy.

The procedures related to internal hernia were distal gastrectomy with Billroth II, Roux-en-Y and uncut Roux-en-Y reconstructions, total gastrectomy with Roux-en-Y reconstruction, and proximal gastrectomy with double tract reconstruction. Internal hernia was diagnosed through surgical exploration or CT, which showed whirling appearance of the mesentery and mesenteric vessels (whirl sign). Patients’ age, sex, body mass index (BMI), history of previous abdominal surgery, mesenteric defect closure (no-closure versus closure), operative approach (open versus laparoscopic), number of ports (multi-port versus single port), type of gastrectomy, type of reconstruction, and route of reconstruction were evaluated for internal hernia.

Categorical data were compared using Pearson’s Chi-squared test, and continuous data were compared using the independent *t* test. Categorical variables were presented as numbers with percentages, and continuous data were expressed as mean ± standard deviation. The risk factors for internal hernia were evaluated using logistic regression analysis. Analyses were conducted using IBM SPSS Statistics version 21 (IBM Co., Armonk, NY, USA) statistical software. A *P* value, less than 0.05 was considered statistically significant.

## Results

Of the 6474 patients, 111 (1.7%) developed internal hernia. The median time to diagnosis of internal hernia was 450 days (3–3398 days).

The characteristics of patients with internal hernia are shown in Table 1. Internal hernia developed in 82 (1.9%) of the 4333 male patients and in 29 (1.4%) of the 2141 female patients (*P* = 0.1).

**Table 1:** Characteristics of patients with internal hernia

Variables	Internal hernia (*N* = 111)	% (Internal hernia/all)
Age (years)	61 ± 14.1	
Sex		
Female	29	1.4% (29/2141)
Male	82	1.9% (82/4333)
BMI (kg/m^2^)		
At the time of gastrectomy	23.5 ± 3.3	
At the diagnosis of internal hernia	20.4 ± 2.7	
BMI change rate (%)		
Increase	3.5 ± 3.7%	4.5% (5^b^/111^a^)
Decrease	11.7 ± 5.9%	95.5% (106^c^/111^a^)
Prior abdominal surgery		
Yes	16	1.4% (16/1177)
No	95	1.8% (95/5297)
Mesenteric defects closure		
Before	88	3.1% (88/2826)
After	23	1.8% (23/1248)

Values are presented as mean ± standard deviation or as % (*N*)

*BMI* body mass index

^a^Number of IH patients

^b^Number of IH patients whose BMI increased

^c^Number of IH patients whose BMI decreased

The mean BMI at the time of gastrectomy and at the diagnosis of internal hernia was 23.5 ± 3.3 kg/m^2^ and 20.4 ± 2.7 kg/m^2^, respectively. The change of the BMI was that 106 (95.5%) of the 111 patients showed decreased BMI and 5 (4.5%) showed increased BMI. The ratios for BMI decrease and increase were 11.7% ± 5.9% and 3.5% ± 3.7%, respectively.

Sixteen (1.4%) of the 1177 patients who had a history of previous abdominal surgery except gastrectomy developed internal hernia, compared to 95 (1.8%) of the 5297 patients without a history of previous abdominal surgery (*P* = 0.3).

In our center, we have standardly closed the mesenteric defects from 2015. Eighty-eight (3.1%) of the 2826 patients without closed defects and 23 (1.8%) of the 1248 patients with closed defects developed internal hernia; the incidence of internal hernia was significantly higher in the non-closure group (*P* = 0.02).

The incidence of internal hernia according to gastrectomy type is shown in Table 2. Fourteen (0.9%) of the 1510 patients who underwent open gastrectomy and 97 (2.0%) of the 4964 patients who underwent laparoscopic gastrectomy developed internal hernia (*P* < 0.01).

**Table 2:** Incidence of internal hernia according to gastrectomy type

Variables	Open (*N* = 1510)	Laparoscopy (*N* = 4964)	
		Multi-port (*N* = 4653)	Single port (*N* = 311)
DG	9/1026 (0.9%)	50/3729 (1.4%)	13/294 (4.4%)
BI	0/571 (0%)	0/1769 (0%)	0/21 (0%)
BII	4/280 (1.4%)	5/511 (1.0%)	0/41 (0%)
RY	2/28 (7.1%)	5/90 (5.6%)	0/32 (0%)
uRY	3/147 (2.0%)	40/1359 (2.9%)	13/200 (6.5%)
TG RY	4/475 (0.8%)	29/638 (4.5%)	0/9 (0%)
PG EG	0/3 (0%)	0/36 (0%)	0/0
PG DTR	1/6 (16.7%)	4/250 (1.6%)	1/8 (12.5%)
*N*	14/1510 (0.9%)	83/4653 (1.8%)	14/311 (4.5%)

*DG* distal gastrectomy, *TG* total gastrectomy, *PG* proximal gastrectomy, *BI* Billroth I, *BII* Billroth II, *RY* Roux-en-Y, *uRY* uncut Roux-en-Y, *EG* esophagogastrostomy, *DTR* double tract reconstruction

Eighty-three (1.8%) of 4653 patients who underwent a multi-port approach and 14 (4.5%) of 311 patients who underwent a single-port approach developed internal hernia (*P* < 0.01).

Totally laparoscopic surgery has been started from 2012 in our center. As getting accustomed to laparoscopic surgery, we started performing this approach because of its less postoperative pain and better cosmetic outcomes than the laparoscopy-assisted approach. Internal hernia developed in 56 (1.9%) of the 3010 patients who underwent laparoscopy-assisted gastrectomy, and 41 (2.1%) of the 1954 patients who underwent totally laparoscopic gastrectomy (*P* = 0.55).

In terms of gastrectomy type, internal hernia developed in 72 (2.7%) of the 2688 patients who underwent distal gastrectomy, in 33 (2.9%) of the 1122 patients who underwent total gastrectomy, and in 6 (2.3%) of the 264 patients who underwent proximal gastrectomy (*P* = 0.81).

Regarding the reconstruction type, internal hernia developed in 9 (1.1%) of the 832 patients with Billroth II reconstruction, in 40 (3.1%) of the 1272 patients with Roux-en-Y reconstruction, in 56 (3.3%) of the 1706 patients with uncut Roux-en-Y reconstruction, and in 6 (2.3%) of the 264 patients with double tract reconstruction. The incidence of internal hernia was significantly higher after uncut Roux-en-Y and Roux-en-Y reconstruction than after Billroth II reconstruction (*P* < 0.01 and *P* < 0.01).

Regarding the route of reconstruction, internal hernia developed in 110 (2.7%) of the 4038 patients with antecolic route, and in 1 (1.7%) of the 60 patients with retrocolic route (*P* = 0.62).

The sites and treatments of internal hernia are shown in Table 3. Fifty-nine (53.2%) of the 111 patients underwent surgical treatments; of these, 33 (55.9%) patients underwent the laparoscopic approach. Of the 59 surgically treated patients with internal hernia, 32 (54.2%) were located at the jejunojejunostomy mesenteric defect and 27 (45.8%) were found at the Petersen’s defect, which is the space between the Roux limb and the transverse mesocolon. There was no internal hernia at transverse colon mesenteric defect among the surgically treated patients. Two (1.8%) patients died due to septic shock caused by bowel strangulation. Nine (8.1%) patients had Clavien–Dindo grade III surgical complications or higher.

**Table 3:** Sites and treatments of internal hernia

Variables	Patients (*N* = 111, %)
Treatment (surgical/conservative)	59/52 (53.2%/46.8%)
Emergency surgery	46/59 (77.9%)
Elective surgery	13/59 (22.1%)
Operative approach	
Open	26/59 (44.1%)
Laparoscopy	33/59 (55.9%)
Bowel resection	
Yes	12/59 (20.3%)
No	47/59 (79.7%)
Sites of hernia (surgically treated)	
Jejunojejunostomy mesenteric defect	32/59 (54.2%)
Petersen’s space	27/59 (45.8%)
Transverse colon mesenteric defect	0/59 (0%)
Mortality	2/111 (1.8%)
Morbidity (≥ CD grade III)	9/111 (8.1%)
Complicated fluid collection	2/111 (1.8%)
Intra-abdominal hematoma	1/111 (0.9%)
SMV thrombosis	1/111 (0.9%)
Bowel ischemia	2/111 (1.8%)
Bowel perforation	1/111 (0.9%)
Sepsis	2/111 (1.8%)

*CD* Clavien–Dindo, *SMV* superior mesenteric vein

Univariate analysis showed that the non-closure of mesenteric defects [odds ratio (OR) 1.72, 95% CI 1.09–2.78, *P* = 0.02] and the laparoscopic approach (OR 2.27, 95% CI 1.27–4.07, *P* = 0.01) were significant risk factors for internal hernia. Multivariate analysis demonstrated that non-closure of mesenteric defects (OR 2.08, 95% CI 1.29–3.33, *P* < 0.01), laparoscopic approach (OR 2.43, 95% CI 1.35–4.38, *P* < 0.01), and totally laparoscopic approach (OR 1.65, 95% CI 1.05–2.61, *P* = 0.03) were independent risk factors for internal hernia (Table 4).

**Table 4:** Multivariate analysis of risk factors for internal hernia

Variables	Univariate analysis		Multivariate analysis	
	OR (95% CI)	*P*	OR (95% CI)	*P*
Age (years)	0.98 (0.97-1.00)	0.07		
Sex		0.42		
Female	0.84 (0.55–1.28)			
Male	1			
BMI (kg/m^2^)	0.95 (0.90–1.01)	0.12		
Prior abdominal surgery		0.29		
Yes	0.75 (0.44–1.28)			
No	1			
Mesenteric defects closure		0.02*		< 0.01*
Before	1.72 (1.09–2.78)		2.08 (1.29–3.33)	
After	1		1	
Operative approach		0.01*		< 0.01*
Laparoscopy	2.27 (1.27–4.07)		2.43 (1.35–4.38)	
Open	1		1	
Type of laparoscopic approach		0.35		0.03*
Totally laparoscopic	1.20 (0.82–1.75)		1.65 (1.05–2.61)	
Laparoscopy-assisted	1		1	
Number of ports		0.77		
Single port	1.12 (0.55–2.24)			
Multi-port	1			
Type of reconstruction		0.19		
Billroth II	0.42 (0.14–1.21)			
Roux-en Y	1.40 (0.59–3.33)			
Uncut Roux-en Y	1.49 (0.63–3.48)			
Double tract reconstruction	1			
Type of gastrectomy		0.88		
Distal gastrectomy	1.20 (0.52–2.79)			
Total gastrectomy	1.26 (0.52–3.05)			
Proximal gastrectomy	1			
Route of reconstruction		0.59		
Antecolic	1.69 (0.23–12.5)			
Retrocolic	1			

*OR* odds ratio, *CI* confidence interval, *BMI* body mass index

*Statistically significant difference (*P* < 0.05)

## Discussion

In this study, we investigated the clinical features and risk factors of internal hernia in patients who underwent gastrectomy for gastric cancer. The closure of Petersen’s, jejunojejunostomy mesenteric and transverse colon mesenteric defects is important for the prevention of internal hernia after gastrectomy.

Recently, laparoscopic surgery has been increasingly performed because of its many advantages compared to open surgery, such as reduced perioperative morbidity and a shortened hospital stay. Several studies on laparoscopic gastrectomy have reported additional benefits, including a better quality of life and an oncologic outcome comparable to open gastrectomy [1–6].

Internal hernia is a critical problem after laparoscopic and open surgery because they might cause life-threatening conditions, such as bowel strangulation or perforation [17, 18]. Furthermore, the incidence of internal hernia has increased to 5–9% after the adoption of laparoscopic surgery [10–14]. Therefore, the evaluation of risk factors is important to prevent this complication. However, there is limited information on internal hernia after gastric cancer surgery.

The present study showed that the overall incidence of internal hernia was 1.7%. Other reported incidences of internal hernia after gastric cancer surgery range from less than 1–7% [4, 14, 15, 19].

Excessive weight loss might be one of the risk factors for internal hernia after gastrectomy. In our study, BMI decreased in 106 (95.5%) of the 111 patients with internal hernia between the time of gastrectomy and the time of diagnosis of internal hernia. The mean BMI reduction rate of these patients was 11.7, which was larger than that reported in other studies (range 6.6–10.8%) [20, 21]. Excessive weight loss is considered one of the risk factors for internal hernia in bariatric surgery [22, 23]. It is presumed that stitches closing the mesentery defects may loosen as the amount of mesenteric fat tissue decreases due to excess body weight loss. This may contribute to the development of internal hernias even after closure of the defects [4, 24, 25].

From studies of gastric cancer surgery, the closure of the mesenteric defects is recommended to reduce the incidence of internal hernia [14, 15, 19]. In this study, the closure of these defects was associated with a significantly lower incidence of internal hernia compared to the non-closure of the defects, and the non-closure of the defects was demonstrated as a risk factor for internal hernia by multivariate analysis. Paroz et al. [7] suggested the closure of all mesenteric defects using a continuous nonabsorbable suture to prevent internal hernia after laparoscopic Roux-en Y gastric bypass (internal hernia incidence: 5.6% absorbable versus 1.3% nonabsorbable; *P* = 0.03).

Several studies have reported that the incidence of postoperative internal hernia was significantly higher after laparoscopic gastrectomy for gastric cancer than after open gastrectomy. Laparoscopy might cause less trauma, leading to fewer adhesions. Hence, the intestine is less adhered to adjacent structures, which may lead to better mobility, thereby enabling it to move into the mesenteric defects [14, 15, 19]. In our study, the incidence of internal hernia was significantly higher in laparoscopic surgery than in open surgery, and the laparoscopic approach was demonstrated as a risk factor for internal hernia by multivariate analysis.

The present study showed higher incidence of internal hernia after single-port surgery compared to multi-port surgery. There were no studies comparing the incidence of internal hernia in regard to the number of ports. In our experience, the outcome might have been attributed to the relatively difficult manipulation of laparoscopic instruments in single-port surgeries. For a free range of motion and reducing the clash between instruments, optimizing the distance between ports is essential, which cannot be fulfilled in single-port surgery [26]. Therefore, meticulous suture to seal the mesenteric defects becomes more demanding for single-port surgery. However, the number of ports was not an independent risk factor for internal hernia by multivariate analysis.

Totally laparoscopic gastrectomy has a higher rate of postoperative internal hernia than laparoscopy-assisted gastrectomy because it is associated with fewer adhesions. In laparoscopy-assisted surgery, a surgeon directly manipulates the bowel through the mini-laparotomy site, causing more adhesions than in totally laparoscopic surgery [19]. Multivariate analysis in this study showed a significantly higher risk for internal hernia in totally laparoscopic surgery than in laparoscopy-assisted surgery.

Kelly et al. [14] reported that internal hernia rarely developed in patients who underwent Billroth II reconstruction than in those who underwent Roux-en Y reconstruction. In the present study, Roux-en Y and uncut Roux-en Y reconstructions showed a significantly higher incidence of internal hernia than Billroth II reconstruction. It might be because Roux-en Y and uncut Roux-en Y reconstruction types have two defects (jejunojejunostomy mesenteric defect and Petersen’s defect) for internal hernia, whereas Billroth II reconstruction has only a Petersen’s defect. However, the reconstruction type was not an independent risk factor for internal hernia by multivariate analysis.

The retrocolic route has three potential spaces for internal hernia, the jejunojejunostomy mesenteric, the transverse colon mesenteric, and Petersen’s defect. For this reason, many surgeons have preferred the antecolic route, which has only two potential spaces, the jejunojejunostomy mesenteric and Petersen’s defect, to reduce the incidence of internal hernia [13, 18, 27, 28]. However, there was not significant difference of the incidence of internal hernia between two groups, and the type of reconstruction route was not an independent risk factor for internal hernia by multivariate analysis.

In this study, 52 (46.8%) of the 111 patients underwent non-surgical treatment for internal hernia. Indication for non-surgical treatment for internal hernia has not been clearly established yet. Lannelli et al. [29] recommended to consider early surgical exploration if there is colicky abdominal pain after laparoscopic Roux-en Y gastric bypass (LRYGB), even in the absence of other symptoms, laboratory and/or radiologic abnormalities. Parakh et al. [30] suggested observation when CT scan was non-diagnostic and abdominal pain improved. Champion et al. [28] recommended surgical exploration when colicky pain persisted or recurred after LRYGB, even if CT finding was non-diagnostic. In our center, the patients without symptoms and bowel ischemia on CT underwent non-surgical management.

There were several limitations to this study. First, it was retrospective; thus, the distribution of background characteristics of the study population was not uniform. Second, since work-up for internal hernia was performed mainly with CT, there are possibilities that the patients, who were asymptomatic without follow-up CT or had false-negative CT findings, were underdiagnosed, leading to an inaccurate estimation of the incidence of internal hernia. Third, this study was conducted at a single center and was limited to the Korean population; thus, our results might not be generalizable to other ethnicities.

## Conclusions

Internal hernia after gastrectomy for gastric cancer is a significant complication because of the possibility of severe morbidities and mortality. The closure of the jejunojejunostomy mesenteric, Petersen’s, and transverse colon mesenteric defects using a nonabsorbable continuous suture is recommended for patients undergoing gastrectomy for gastric cancer to reduce the incidence of internal hernia.
